# Adiponectin and HMW Oligomers in Relation to Inflammatory Markers in Crohn’s Disease Patients

**DOI:** 10.3390/biomedicines13020273

**Published:** 2025-01-23

**Authors:** Maurizio Marra, Marta Mallardo, Ersilia Nigro, Iolanda Cioffi, Camilla Leo, Alessia Dalila Guarino, Fabiana Castiglione, Fabrizio Pasanisi, Aurora Daniele

**Affiliations:** 1 Department of Clinical Medicine and Surgery, University of Naples “Federico II”, Via Pansini 5, 80131 Naples, Italy; maurizio.marra@unina.it (M.M.); iolanda.cioffi@unimi.it (I.C.); fabrizio.pasanisi@unina.it (F.P.); 2 Department of Molecular and Biotechnological Medicine, University of Naples “Federico II”, 80138 Naples, Italy; marta.mallardo@unicampania.it (M.M.); aurora.daniele@unina.it (A.D.); 3 CEINGE-Biotechnologies Advances S.c.a r.l., Via G. Salvatore 486, 80145 Naples, Italy; 4 Dipartimento di Scienze e Tecnologie Ambientali, Biologiche, Farmaceutiche, Università della Campania “Luigi Vanvitelli”, Via Vivaldi 43, 81100 Caserta, Italy; 5 Division of Human Nutrition, Department of Food, Environmental and Nutritional Sciences—DEFENS, Università Degli Studi di Milano, Celoria 2, 20133 Milan, Italy; 6 Gastroenterology Unit, Department of Clinical Medicine and Surgery, “Federico II” University, 80131 Naples, Italy; camilla.leo@unina.it (C.L.); alessia.guarino@unina.it (A.D.G.); fabiana.castiglione@unina.it (F.C.)

**Keywords:** Crohn’s disease (CD), adiponectin/HMW adiponectin, inflammation, CDAI

## Abstract

**Background/Objectives:** Crohn’s disease (CD), a chronic inflammatory gastrointestinal illness, is the result of genetics and environmental factors. Adipose tissue has recently been found to play a significant role in CD. **Methods**: here, we examined the relationship between adiponectin, HMW oligomers, and CD in 92 patients. **Results**: first, we verified that the patients’ therapies had no influence on the nutritional and biochemical variables. Correlation analysis between HMW adiponectin and nutritional parameters found no relationship; therefore, we investigated its relationship with CD severity and inflammatory markers. Based on adiponectin, we divided patients into tertiles and found that CDAI was lowest in the second and third tertile [I: <19.3 (*n* = 31); II: 19.3–22.2 (*n* = 31); III: >22.2 (*n* = 30)]. Furthermore, IL-6 and CRP were higher in the second and third tertile, while lymphocytes were lower in the second and third tertile. Correlation analysis showed that both adiponectin and HMW oligomers were inversely related to lymphocytes and directly related to CRP. A ROC curve evidenced that patients carrying adiponectin values ≤ 19 performed well in predicting worse CDAI and lymphocyte values (AUCs: 0.826 and 0.806). Next, we divided CD patients into tertiles based on HMW adiponectin, finding that IL-6 was highest in the second and third tertile. Lymphocytes were lowest in the third tertile while CRP values were substantially higher. **Conclusions**: altogether, these data suggest a biological role for adiponectin and HMW oligomers in CD severity and inflammatory status. However, the molecular effects related to adiponectin in CD remain unclear and further studies are needed to clarify its applicability as a biomarker.

## 1. Introduction

Crohn’s disease (CD) is a chronic inflammatory bowel disease (IBD), characterized by a relapsing–remitting progression [1], whose incidence is increasing world-wide, largely affecting the population of young adults, with significant psychological and social implications [2].

The molecular pathogenetic mechanisms underlying CD are not yet fully characterized but a combination of genetics, environmental factors, and immunological abnormalities are important components of its etiology and development [3]. Strong evidence suggests that patients with CD may be at higher risk of developing metabolic diseases that impact on the risk of complications [4]. Furthermore, several recent studies have attributed a significant role to visceral adipose tissue in the development and progression of inflammatory disorders such as CD; on the one hand, it has been reported that the ratio of intra-abdominal fat to total abdominal fat seems to be higher in CD patients than in normal controls [5,6], and on the other hand, a higher percentage of visceral fat has been associated with a higher rate of postoperative disease recurrence [5,6]. Furthermore, the endocrine functions of adipose tissue appear inseparably linked to CD activity and general outcome [7]. For example, data in the literature report that the serum concentrations of several adipokines such as leptin, adiponectin, and resistin are correlated with disease severity, body composition, and glycemic homeostasis in CD while, on the contrary, other studies report no regulation of adipokine levels, making it difficult to draw conclusions [8].

Among the others, adiponectin is one of the most abundant adipokines in the serum with a routine range of 3–30 μg/mL, corresponding to approximately 0.01–0.05% of the total serum protein [9,10,11,12]. Structurally, adiponectin belongs to the soluble defense collagen superfamily with substantial homology with collagen X and VIII and complementing factor C1q [13]. The protein is composed of a signal peptide, a variable N-terminal domain, a collagenous domain comprising 22 Gly-Xaa-Yaa repeats, and a C-terminal C1q-like globular domain [13]. Importantly, adiponectin assembles into structures of different molecular weights: trimer low-molecular-mass (LMW), hexamer medium-molecular-mass (MMW) and multimer (HMW). The trimeric adiponectin, LMW isoform, is formed via hydrophobic interactions within the globular heads of adiponectin and stabilized by the non-covalent interactions of the collagenous domains in a triple-helix stalk. The hexameric adiponectin, MMW oligomeric complex, is formed through the disulfide bond-mediated self-association of two homotrimers [13]. The structural characteristics of HMW adiponectin remain poorly characterized. Nevertheless, specific functional roles have been outlined for the different oligomers, and it is clearly demonstrated that HMW are the most biologically relevant oligomers [14].

In obesity, hypoadiponectinemia is associated with insulin resistance, cardiovascular diseases, and others [10,11]. In other different diseases, adiponectin and its oligomers are dysregulated, as they act as anti-inflammatory mediators [15]. Regarding CD, adiponectin is thought to have a major role in the development and severity of the disease [16] since, although human studies have produced conflicting results, altogether they support the hypothesis that this adipokine plays a central role in intestinal homeostasis [17,18].

In this scenario, we analyzed the adiponectin serum levels in 92 CD patients, according to disease activity and inflammatory level. The oligomerization state of adiponectin as well as its relationship with multiple indicators of nutritional status and inflammatory biomarkers were also evaluated. We looked into the potential relations between pro-inflammatory cytokines, malnutrition risk screened by the CONUT score, and body composition with adiponectin to clarify the potential use of adiponectin as a biomarker of CD and to take a step towards the understanding of its biological role in CD.

## 2. Materials and Methods

### 2.1. Design and Study Population

This is a retrospective analysis that includes a cohort of ninety-two adult (53 females, 39 males) consecutive CD outpatients recruited at the Department of Clinical Medicine and Surgery, Federico II University Hospital, Naples (Italy), between July 2016 and March 2018 [19]. The inclusion and exclusion criteria have been previously described [20]. Clinically, disease activity was assessed using the Crohn’s Disease Activity Index (CDAI), categorizing patients into active (CDAI ≥ 150) and quiescent (CDAI < 150) phases. Demographic data, disease duration, prior surgeries, and disease location and behavior (according to the Montreal classification) were also collected [21]. The study adhered to the Declaration of Helsinki and was approved by the Federico II Ethical Committee (No. 102/16) and registered on clinicaltrials.gov as NCT03054935 (November 2016). All participants provided written informed consent before enrollment.

### 2.2. Anthropometric, Body Composition Parameters, and Muscle Strength Measurement

Anthropometric parameters included measurements of weight and height; the calculation of BMI, and of percentage of fat mass (FM) and free fat mass (FFM) were determined as previously reported [19]. The BIA variables considered were R and Xc, while PhA was calculated as arc tangent Xc/R × 180°/π. FFM and FM were estimated using the predictive equations developed by Sun [22].

### 2.3. Biochemical Parameters

Blood samples were collected to assess various biomarkers related to nutritional status, including albumin (g/dL), hemoglobin (Hb) (g/dL), total cholesterol (mg/dL), total lymphocytes (10−3/mL), prealbumin (g/L), pseudocholinesterase (UI), alpha-2 fraction (%), C-reactive protein (CRP) (mg/L), fibrinogen (mg/dL), total protein (g/dL), transferrin (g/dL), and ferritin (ng/dL). These parameters were analyzed at the Federico II University Hospital using standardized techniques [20].

### 2.4. Nutritional Screening Risk Tool: CONUT Score

The CONUT score was calculated as previously described [23]. Patients were classified as having normal (0–1), light (2–4), moderate (5–8), and severe (9–12) risk of malnutrition [23].

### 2.5. Total and HMW Adiponectin Determination

Total serum adiponectin concentration was estimated by an enzyme-linked immunosorbent assay (ELISA), as previously reported [24]. Additionally, circulating HMW adiponectin was measured with a specific human ELISA kit according to the manufacturer’s instructions (R&D Systems, Minneapolis, MN, USA).

### 2.6. Adiponectin Oligomerization State Analysis

The adiponectin oligomerization state was analyzed by Western blotting. Briefly, serum samples were quantified for total proteins by Bradford’s method (Bio-Rad, Hercules, CA, USA) and Western blotting was performed as previously described [25]. The blots were scanned by using a ChemiDoc MP imaging system (Bio-Rad, CA, USA) and analyzed by densitometry with ImageJ software version 1.54m 5 December 2024 (http://imagej.net/ij/). Samples were analyzed and tested two times in duplicate.

### 2.7. Statistical Analysis

Statistical analysis was conducted using SPSS ver. 22.0 (IBM Corporation, Inc., Chicago, IL, USA). The data are presented as mean ± standard deviation [SD], unless stated otherwise. The normality of variables was assessed using the Kolmogorov–Smirnov test and the Shapiro–Wilk test. The differences between sexes were analyzed by an independent T-test, while the differences between tertiles were analyzed using a one-way ANOVA with Tukey post hoc. Linear correlation was utilized to evaluate associations between variables. A *p* value < 0.05 was considered statistically significant. Receiver-operating characteristic (ROC) curves were used to identify the parameter that was most associated with 1° tertile of adiponectin concentration.

## 3. Results

### 3.1. Study Population

Measurements of anthropometric characteristics of 92 CD patients were performed, including weight and height, calculation of body mass index (BMI), and determination of percentage of fat mass (FM) and free fat mass (FFM) (see Table 1). From the analysis and comparison, BMI, FFM, Phase Angle, and HGS were statistically higher in males than females. The disease phenotype was classified according to the Montreal classification, revealing that CD was diagnosed mainly between the ages of 17 and 40 years, localized in the ileo-colon with a structuring phenotype.

Previous surgery due to disease complications was found in 47 patients. A total of 45 patients were clinically quiescent (CDAI < 150), while 47 showed mild to moderate disease activity (150 > CDAI < 450). Of the patients, 31.5% were not on medications, whilst 46.74% were on biologics, 9.8% on immunosuppressives, and 12% were taking amino salicylic acid. The CONUT scores were as following: 35.9% CONUT score < 2; 53.3% < 4 CONUT score > 2; 9.8% 8 < CONUT score > 4; 1.1% CONUT score > 10.

Table 2 shows some of the inflammatory parameters measured and the CDAI disease severity index in the study population: no statistical differences were found in all the parameters considered.

### 3.2. Total Adiponectin and HMW Oligomers Are Related to Disease Severity and Lymphocytes

Next, to better understand the distribution of data, we used tertiles to split data into three groups and to use them in comparisons with other variables. Accordingly, we divided patients into tertiles based on adiponectin concentration (see Table 3). Interestingly, only in patients belonging to the second and third tertiles did we find statistical differences; in fact, while CDAI and lymphocytes were statistically lower, IL6 and CRP levels were statistically higher.

Since data in the literature have shown that HMW oligomers are the most active form of adiponectin, we divided CD patients into tertiles based on their concentration (Table 4). As for total adiponectin, we found that IL6 levels were higher in patients within the second and the third tertile. Lymphocytes were lower in the third tertile while CRP values were substantially higher.

**Figure 1:**
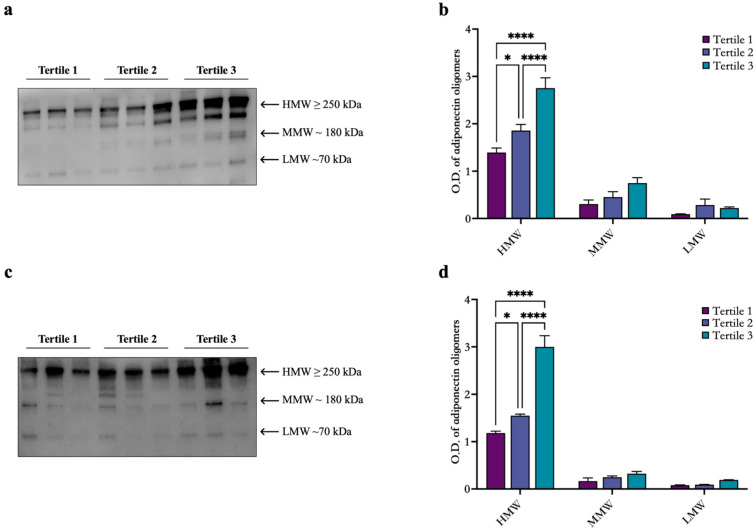
Oligomeric distribution of adiponectin in CD patients. Representative Western blot images for HMW, MMW, and LMW adiponectin oligomers in the serum of male (**a**) and female (**c**) patients diagnosed with CD. (**b**,**d**) Graphical representation of pixel quantization of analyzed CD patients. * *p* < 0.05; **** *p* < 0.0001.

Additionally, we analyzed the adiponectin oligomers’ distributions by Western blotting (WB). The results of WB analysis showed three bands corresponding to HMW (≥250 kDa), MMW (≥180 kDa), and LMW (≥70 kDa) in CD patients (Figure 1). Interestingly, the densitometric analysis (Figure 1b,d) highlights a significant increase in HMW oligomers among adiponectin tertiles.

### 3.3. Correlation Analysis of Total Adiponectin and HMW Oligomers with Inflammatory Markers

**Figure 2:**
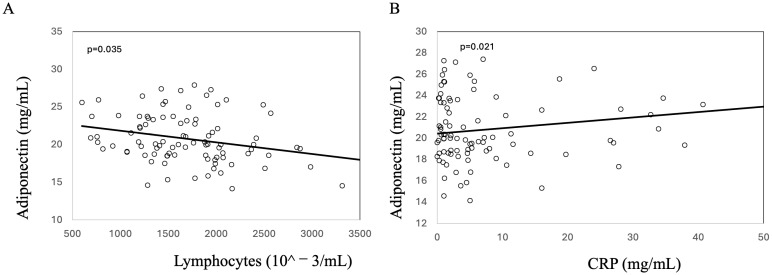
Linear correlation between adiponectin and lymphocytes (**A**) CRP (**B**) in 92 patients with Crohn’s disease.

Successively, we performed a correlation analysis to further verify the association of adiponectin with some inflammatory parameters. The analysis showed that adiponectin is inversely related to lymphocytes (Figure 2A) and directly related to CRP (Figure 2B).

**Figure 3:**
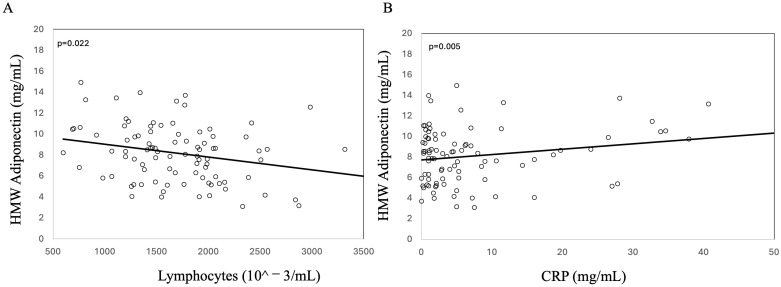
Linear correlation between HMW adiponectin and lymphocytes (**A**) and CRP (**B**) in 92 patients with Crohn’s disease.

Then, we analyzed the relationship between HMW adiponectin and clinical parameters, finding that HMW adiponectin was inversely related to lymphocytes (Figure 3A) and directly related to CRP (Figure 3B).

### 3.4. ROC Analysis

**Figure 4:**
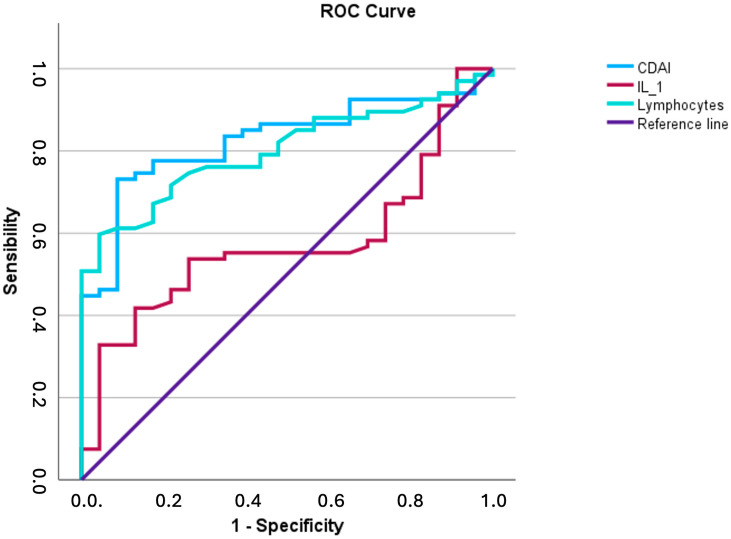
The receiver-operating characteristic (ROC) curve indicated that CDAI and lymphocytes performed best (AUCs: 0.826 and 0.806), while IL1-β proved to be an inadequate indicator (AUCs: 0.587) to predict patients with adiponectin ≤ 19.6 μg/mL.

For this analysis, three disease severity and inflammatory parameters (lymphocytes, CDAI, IL-1β) were selected because they occur in the total sample as abnormalities in more than 10% patients. Patients were divided into two groups based on the adiponectin values (Group A ≤ 19.6 − 1° tertile; Group B > 19.6 − 2° and 3° tertile). The AUC (Area Under Curves) was used to determine which of these three indicators could better identify patients belonging to Group A. CDAI and lymphocytes performed best (AUCs: 0.826 and 0.806), whereas IL1-β proved to be an inadequate indicator (AUCs: 0.587) (Figure 4).

## 4. Discussion

Adiponectin is a well-characterized collagenous adipokine with multiple biological functions including anti-inflammatory functions in many peripheral tissues and organs [26,27,28]. In particular, adiponectin has been shown to play a protective role in many gastrointestinal diseases, where it prevents chemokine production and inflammatory responses by inhibiting macrophage infiltration and release of pro-inflammatory cytokines [25,29,30]. Regarding the specific involvement of adiponectin in CD, different studies have yielded conflicting results.

In this study, we examined the potential relationship between adiponectin, HMW oligomers, and Crohn’s disease. For this purpose, we considered 92 patients with CD, characterized for nutritional and inflammatory status as well as for body composition, who were part of ancillary studies [19,20,31,32]. First, as a premise for this study, we verified whether the pharmacological treatments to which the patients of the study had been subjected had an influence on the nutritional and biochemical variables considered in our study, failing to find any. We then performed correlation analyses between adiponectin, HMW oligomers and nutritional and/or anthropometric parameters (FM, PhA, FFM, HSG), failing to find any relationship. Therefore, since adiponectin does not seem to be related to metabolic parameters nor to body composition, we focused on investigating its potential relationship with CD severity and inflammatory markers. Interestingly, we found that total adiponectin levels are inversely distributed in CD patients in relation to CDAI disease severity. Indeed, higher levels of adiponectin were associated with lower CDAI. In support of our evidence, previously, serum adiponectin expression was found to be lower in active CD patients compared to controls, although no differences were observed when comparing the active and remission CD patients [33]. In another study, significantly increased tissue levels and the release of adiponectin were reported in hypertrophied CD patients compared to CD patients, suggesting a defective regulation of anti-inflammatory pathways in CD severe patients [34]. Furthermore, in a subsequent study, serum adiponectin levels were found to be increased or unchanged in CD patients [17]. The differences between the various published data and our results could be partly explained by the different sizes of the populations studied, different disease severity among analyzed patients, treatment status of patients, or inadequate controls.

The molecular mechanisms underlying the relationships between adiponectin and Crohn’s disease are far from being clearly elucidated. However, adipose tissue has been revealed in recent years as a crucial endocrine regulator that, by secreting adipokines, exerts pro- or anti-inflammatory activities by secreting adipokines such as IL-6, tumor necrosis factor α (TNF-α), and plasminogen activator inhibitor type 1 [35]; furthermore, more recently, the role of ghrelin and obestatin have been investigated, demonstrating that IBD patients with active disease have higher levels of ghrelin and also that the obestatin/ghrelin ratio might be a biomarker for disease activity assessment [36]. While the balance between pro- and anti-inflammatory adipokines guarantees homeostasis in a normal metabolic status, this balance is moved toward a pro-inflammatory state in the case of pathological conditions. Numerous studies underlined the pivotal role of adiponectin regulation in the above-mentioned balance; indeed, the loss of the anti-inflammatory role of adiponectin seems to intervene in the etiopathogenesis of several disorders, including bowel diseases.

In this scenario, we analyzed inflammatory parameters, observing that higher levels of adiponectin are associated with lower lymphocytes values and with higher levels of IL-6 and CRP inflammatory markers. The relationship we found among adiponectin, IL-6, and CRP suggests that this adipokine participates in the inflammatory milieu of CD, thus representing a candidate marker of disease severity. Clinical studies on CD patients reported a direct association between IL-6 and the disease severity index CDAI and a direct relation between CRP values and a worse CD phenotype [19,20,35,36]. Such evidence has been enough to propose IL-6 and CRP as markers for disease activity and severity [37] but the identification of multiple biomarkers useful in the classification of patients according to disease activity and severity needs to be urgently implemented. In this context, our data support the suitable of adiponectin as an additional index for disease severity and for the inflammatory status. It is noteworthy, however, that in contrast with our data, no correlation between adiponectin values and CRP has been previously reported in CD patients [38].

Regarding the inverse correlation between adiponectin and lymphocytes, we do believe that these data further support the hypothesis of this adipokine as an additional signature molecule for CD [39]. Lymphocytes are immune cells that play a crucial role in CD, where they accumulate in the gut mucosa, activating the expression of endothelial adhesion molecules, which in turn lead to the development of intestinal lesions. Furthermore, a clinically relevant role for CD8 T cell subset exhaustion in CD has been revealed [40].

The ROC curve analysis performed, considering lymphocytes, CDAI, and IL-1β, evidenced that CDAI and lymphocytes indices performed best to predict low adiponectin values, strengthening the hypothesis of a role of adiponectin in modulating inflammation levels in CD and the chance of listing adiponectin in the arsenal of novel CD biomarkers.

Considering the functional meaning of the adiponectin oligomerization state, we further analyzed CD patients for the oligomer’s distribution. Indeed, oligomeric states of adiponectin, such as trimers, hexamers, and octadecamers, are specifically involved in different biological functions such as metabolism, immunity, inflammation, and cellular homeostasis; mutations affecting adiponectin assembly cause a marked reduction in high molecular weight forms and, in turn, reduce its biological activity in vivo. To our knowledge, only one study has analyzed HMW adiponectin oligomers, those oligomers bearing the highest biological activity [41], finding reduced levels in CD patients. As for total adiponectin, we found that IL6 levels are higher in patients within the second and the third tertile, while lymphocytes were lower in the third tertile and CRP values were substantially higher. Additionally, HWM oligomers are directly related to CRP while inversely related with lymphocytes, further supporting the hypothesis that the regulation of both adiponectin and HMW levels is a response to the inflammatory state established in CD. Such evidence suggests that the most active adiponectin oligomers specifically participate in the control of the inflammatory milieu of CD.

Altogether, our data substantially support the involvement of adiponectin in intestinal inflammation. Here, a bidirectional interaction between immune cells and adiponectin is established, resulting in the modulation of immune functions and regulation of adiponectin levels. Furthermore, the relationship we found among adiponectin IL-6 and CRP confirms that this adipokine participates in the inflammatory milieu present in CD patients. However, some limitations of this study need to be highlighted. First, this is a retrospective analysis performed in a specific selected sample of patients with CD, and probably not fully representative of the disease severity and progression. In addition, the study design adopted (cross-sectional) lacked a matched control group, not allowing us to establish the usefulness of adiponectin as a diagnostic disease biomarker. Finally, the use of CDAI for defining disease activity, albeit conventionally accepted, might not be totally accurate, and additional parameters for the evaluation of disease severity need to be included. Furthermore, recent studies have shown a modulation of several transcripts such as miRNAs and some of their target genes in mesenteric adipose tissue of Crohn’s patients [42], but whether adiponectin is associated with these miRNAs remains to be verified.

## 5. Conclusions

In conclusion, the results of this study suggest that adiponectin and its HMW oligomers play an important role in the inflammatory state that characterizes CD patients. Adiponectin could therefore represent a new biomarker to monitor the inflammatory state of CD patients. From a functional point of view, we can speculate that adiponectin levels are regulated in response to the inflammatory state of CD, participating in the induction of a biological response aimed at solving the inflammatory state typical of the disease, especially in active patients. Thus, the molecular effects underlying adiponectin action in CD remain unclear; therefore, further studies are needed to clarify its applicability as a biomarker in this disease. Furthermore, larger prospective studies are needed to verify the feasibility and reliability of these data in different contexts.

## Figures and Tables

**Table 1:** Anthropometric characteristics and handgrip strength in the 92 patients with Crohn’s disease.

		Males (*n* = 53)			Females (*n* = 39)			All (*n* = 92)		
Age	years	39.2	±	12.9	40.0	±	15.1	39.5	±	13.8
Weight	kg	69.8	±	9.4 *	59.3	±	11.9	65.3	±	11.7
Stature	cm	172	±	6 *	159	±	6	167	±	9
BMI	kg/m^2^	23.5	±	2.9	23.4	±	4.4	23.5	±	3.6
FFM	kg	56.4	±	5.4 *	39.7	±	6.0	49.3	±	10.1
FM	kg	13.4	±	7.5 *	19.6	±	8.5	16.0	±	8.4
FM	%	18.4	±	8.4 *	32.0	±	8.5	24.2	±	10.8
Phase Angle	degrees	6.81	±	0.91 *	5.67	±	0.59	6.32	±	0.97
HGS	kg	36.7	±	7.2 *	18.4	±	4.7	28.8	±	11.0

* = *p* < 0.005; BMI = body mass index; FFM = fat free mass; FM = fat mass; HGS = handgrip strength.

**Table 2:** Systemic inflammatory markers in 92 patients with Crohn’s disease.

	Males (*n* = 53)			Females (*n* = 39)			All (*n* = 92)		
IL-1β (pg/mL)	0.230	±	0.355	0.231	±	0.233	0.230	±	0.308
IL-6 (pg/mL)	4.69	±	5.3	5.09	±	6.45	4.86	±	5.79
TNFα (pg/mL)	11.4	±	5.5	11.1	±	4.3	11.3	±	5.00
Lymphocytes (×10^9^/L)	1728	±	5.90	1717	±	551	1724	±	571
Fibrinogen (mg/dL)	353	±	84	391	±	93	369	±	89
CRP (mg/dL)	6.48	±	9.45	7.03	±	9.0	6.71	±	9.26
CDAI score	134	±	78	159	±	76	145	±	78

**Table 3:** Anthropometric characteristics, biochemical parameters, and the CDAI disease severity in 92 patients with Crohn’s disease divided into adiponectin tertiles.

		Adiponectin <19.3 (*n* = 31)			Adiponectin 19.3–22.2 (*n* = 31)			Adiponectin >22.2 (*n* = 30)		
		1° Tertile			2° Tertile			3° Tertile		
***CDAI*** *	score	175	±	73	122	±	82	149	±	82
Age	years	40.3	±	14.9	38.4	±	13.0	39.2	±	14.2
Weight	kg	63.8	±	14.0	67.5	±	9.7	62.4	±	8.5
BMI	kg/m^2^	23.2	±	3.6	23.8	±	2.6	21.9	±	2.7
FFM	kg	46.9	±	11.5	52.0	±	9.3	50.0	±	9.8
FAT	kg	17.0	±	7.4	15.5	±	7.5	12.4	±	7.6
FAT	%	26.4	±	9.2	22.9	±	10.3	19.9	±	11.7
Phase Angle	degrees	6.05	±	0.85	6.55	±	0.94	6.23	±	0.97
HGS	kg	29.6	±	12.4	32.3	±	12.1	30.8	±	10.5
IL-1β		0.290	±	0.270	0.202	±	0.201	0.568	±	1.567
***IL-6*** ***^#^***		6.72	±	8.54	6.11	±	12.1	7.85	±	10.0
TNFα		11.9	±	5.1	11.6	±	5.3	11.4	±	4.8
***Lymphocytes*** *		1900	±	576	1624	±	570	1573	±	531
***CRP*** *		6.75	±	8.53	7.22	±	9.61	13.0	±	22.9

* *p* < 0.05 between tertiles; *^#^ p* < 0.10 between tertiles.

**Table 4:** Anthropometric characteristics, biochemical parameters, and the CDAI disease severity in 92 patients with Crohn’s divided into HMW adiponectin tertiles.

		HMW Adiponectin <6.82 (*n* = 31)			HMW Adiponectin 6.82–9.24 (*n* = 31)			HMW Adiponectin >9.24 (*n* = 30)		
		1° Tertile			2° Tertile			3° Tertile		
CDAI	score	136	±	82	158	±	63	153	±	98
Age	years	39.0	±	14.0	40.6	±	13.8	38.3	±	14.2
Weight	kg	70.9	±	10.1	60.5	±	9.3	62.4	±	11.2
BMI	kg/m^2^	24.3	±	2.8	22.3	±	2.9	22.3	±	3.1
FFM	kg	54.7	±	8.2	45.9	±	10.2	48.1	±	10.7
FAT	kg	16.1	±	8.4	14.6	±	6.8	14.3	±	7.7
FAT	%	22.2	±	10.6	24.3	±	10.6	22.8	±	11.1
Phase Angle	degrees	6.7	±	0.83	6.1	±	0.99	6.0	±	0.85
HGS	kg	36.2	±	8.9	27.6	±	12.3	29.1	±	11.7
IL-1β		0.275	±	0.414	0.532	±	1.512	0.242	±	0.234
***IL-6*** ***^#^***		5.33	±	6.81	9.04	±	15.1	6.26	±	6.09
TNFα		11.5	±	6.5	12.0	±	4.4	11.4	±	3.8
***Lymphocytes*** *		1801	±	513	1806	±	611	1486	±	546
***CRP*** *		5.18	±	6.90	6.32	±	6.25	15.5	±	23.8

* *p* < 0.05 between tertiles; *^#^ p* < 0.10 between tertiles.

## Data Availability

Data are contained within the article.
